# FDG-PET/CT(A) imaging in large vessel vasculitis and polymyalgia rheumatica: joint procedural recommendation of the EANM, SNMMI, and the PET Interest Group (PIG), and endorsed by the ASNC

**DOI:** 10.1007/s00259-018-3973-8

**Published:** 2018-04-11

**Authors:** Riemer H. J. A. Slart, Writing group, Riemer H. J. A. Slart, Andor W. J. M. Glaudemans, Panithaya Chareonthaitawee, Giorgio Treglia, Florent L. Besson, Thorsten A. Bley, Daniel Blockmans, Ronald Boellaard, Jan Bucerius, José Manuel Carril, Wengen Chen, Maria C. Cid, Bhaskar Dagupta, Sharmila Dorbala, Olivier Gheysens, Fabien Hyafil, Shaifali Jain, Thorsten Klink, Conny J. van der Laken, Francisco Lomeña, Michela Massollo, Sergio Prieto-González, Raashid Luqmani, Anne Roivainen, Carlo Salvarani, Antti Saraste, Michael Schirmer, Hein J. Verberne, Annibale Versari, Alexandre E. Voskuyl, Martin A. Walter, Dario Camellino, Elisabeth Brouwer, Marco A. Cimmino, Reviewer group, Aiden Abidov, Denis Agostini, Rob S. Beanlands, Roberto C. Delgado-Bolton, Andrew J. Einstein, Alessia Gimelli, Edward J. Miller, Roberto Sciagrà, Alberto Signore, Members of EANM Cardiovascular, Riemer H. J. A. Slart, Jan Bucerius, Fabien Hyafil, Hein J. Verberne, Denis Agostini, Alessia Gimelli, Roberto Sciagrà, Members of EANM Infection & Inflammation, Andor W. J. M. Glaudemans, Giorgio Treglia, Olivier Gheysens, Annibale Versari, Alberto Signore, Members of Committees, SNMMI Cardiovascular, Panithaya Chareonthaitawee, Wengen Chen, Sharmila Dorbala, Members of Council, PET Interest Group, Andor W. J. M. Glaudemans, Giorgio Treglia, Florent L. Besson, Thorsten A. Bley, Daniel Blockmans, Ronald Boellaard, José Manuel Carril, Maria C. Cid, Bhaskar Dagupta, Olivier Gheysens, Fabien Hyafil, Shaifali Jain, Thorsten Klink, Conny J. van der Laken, Francisco Lomeña, Michela Massollo, Sergio Prieto-González, Raashid Luqmani, Anna Roivainen, Carlo Salvarani, Antti Saraste, Michael Schirmer, Annibale Versari, Alexandre E. Voskuyl, Martin A. Walter, Dario Camellino, Elisabeth Brouwer, Marco A. Cimmino, Members of ASNC, Panithaya Chareonthaitawee, Sharmila Dorbala, Aiden Abidov, Rob S. Beanlands, Andrew J. Einstein, Edward J. Miller, EANM Committee Coordinator, Roberto C. Delgado-Bolton

**Affiliations:** University of Groningen, Medical Imaging Center, Department of Nuclear Medicine & Molecular Imaging, University Medical Center Groningen, Hanzeplein 1, P.O. Box 30001, 9700 RB Groningen, The Netherlands

**Keywords:** Large vessel vasculitis, Polymyalgia rheumatica, FDG-PET/CT(A), Imaging procedure

## Abstract

Large vessel vasculitis (LVV) is defined as a disease mainly affecting the large arteries, with two major variants, Takayasu arteritis (TA) and giant cell arteritis (GCA). GCA often coexists with polymyalgia rheumatica (PMR) in the same patient, since both belong to the same disease spectrum. FDG-PET/CT is a functional imaging technique which is an established tool in oncology, and has also demonstrated a role in the field of inflammatory diseases. Functional FDG-PET combined with anatomical CT angiography, FDG-PET/CT(A), may be of synergistic value for optimal diagnosis, monitoring of disease activity, and evaluating damage progression in LVV. There are currently no guidelines regarding PET imaging acquisition for LVV and PMR, even though standardization is of the utmost importance in order to facilitate clinical studies and for daily clinical practice. This work constitutes a joint procedural recommendation on FDG-PET/CT(A) imaging in large vessel vasculitis (LVV) and PMR from the Cardiovascular and Inflammation & Infection Committees of the European Association of Nuclear Medicine (EANM), the Cardiovascular Council of the Society of Nuclear Medicine and Molecular Imaging (SNMMI), and the PET Interest Group (PIG), and endorsed by the American Society of Nuclear Cardiology (ASNC). The aim of this joint paper is to provide recommendations and statements, based on the available evidence in the literature and consensus of experts in the field, for patient preparation, and FDG-PET/CT(A) acquisition and interpretation for the diagnosis and follow-up of patients with suspected or diagnosed LVV and/or PMR. This position paper aims to set an internationally accepted standard for FDG-PET/CT(A) imaging and reporting of LVV and PMR.

**Electronic supplementary material:**

The online version of this article (10.1007/s00259-018-3973-8) contains supplementary material, which is available to authorized users.

## Introduction

Large vessel vasculitis (LVV) is defined as a disease mainly affecting the large arteries, with two major variants, Takayasu arteritis (TA) and giant cell arteritis (GCA) [1]. Vasculitis can be distributed locally in the branches of the internal and external carotid artery or the aorta and its main branches more centrally in the thorax. TA and GCA are different diseases with different age of onset, ethnic distribution, immunogenic background [2], and distribution and therapy response [3, 4] of the affected arteries. GCA and TA also show some overlap with regard to the histopathology of arterial lesions, reflecting shared pathways in tissue inflammation [5, 6]. Clinically, GCA and polymyalgia rheumatica (PMR) belong to a disease spectrum, and both often coexist in the same patient. Nearly half of patients with GCA have evidence of PMR, while approximately 20% of patients with PMR have concomitant GCA [7, 8], although the frequency of GCA in PMR (either by biopsy or imaging) may vary, depending on the cohort selection criteria.

FDG-PET/CT is a functional imaging technique which is an established tool in oncology, and has also demonstrated a role in the field of inflammatory diseases. FDG-PET is based on the ability to detect enhanced glucose uptake from high glycolytic activity of inflammatory cells in inflamed arterial walls and synovia/bursa [9]. Thereby, it can identify the presence of systemic LVV in patients with GCA and TA, and it can also show inflammation of peri-articular and extra-articular synovial structures in the case of PMR. Approximately 20% of patients with apparently isolated PMR show LVV on FDG-PET/CT [10], and this percentage can be even higher, depending on the presence of LVV symptoms [11–13]. It is important to realize that a negative temporal artery biopsy, an ultrasonography without a halo sign, or magnetic resonance imaging (MRI) without aortic wall thickening or edema does not definitively exclude the presence of LVV and should therefore not limit the use of FDG-PET/CT when LVV is clinically suspected [14, 15]. Furthermore, there is substantial variation in the type of vessels involved (i.e. aortic and cranial large vessels) [16], which can be detected by FDG-PET, given its whole-body scan nature, with the exception of the temporal artery, due to the high physiological FDG uptake in the brain and limited resolution of the camera system. In addition, FDG-PET may assist in the differential diagnosis between PMR and elderly-onset rheumatoid arthritis (EORA) or spondyloarthritis [8], according to the location of inflammation (articular, capsular, or extracapsular). In patients with fever of unknown origin (FUO), when the diagnosis of systemic LVV is ruled out, FDG-PET/CT results enable the identification of other causes of the inflammatory process, including oncological diseases, in the majority of cases. Functional FDG-PET combined with anatomical CT angiography, FDG-PET/CT(A), may be of synergistic value for optimal diagnosis, disease activity monitoring, and evaluation of damage progression in LVV [17]. The main limitation of FDG-PET/CT(A) to becoming a standardized diagnostic tool is the lack of an internationally accepted definition of vascular inflammation and/or PMR, based on the intensity and pattern of the glucose analogue uptake. Also, FDG-PET/CT is not disease specific and is primarily developed to diagnose malignant and infectious/inflammatory diseases. Results have to be interpreted with caution as inflammatory/metabolic changes in the arterial wall usually precede anatomic changes [18–23]. Furthermore, whereas increased FDG uptake is mainly seen in active disease processes, information of advanced stages, for example calcification in chronic or past inflammation, is mainly provided by morphological imaging [24]. Atherosclerosis activity may also interfere with the FDG-PET signal in patients with LVV [25]. Finally, the instigating inflammatory process may have subsided, leaving residual arterial stenosis or aortic aneurysms for which FDG-PET is not the best imaging option.

In nuclear medicine, procedural guidelines for FDG-PET imaging have been published for both cancer [26] and infection/inflammation [27]. However, LVV and PMR are distinct disease entities, which require a specific technical approach. The interpretation of FDG-PET images for LVV can be challenging, and there is currently no consensus on how to interpret the images in the setting of LVV. Furthermore, as previously described, FDG uptake has been demonstrated to respond to glucocorticoids (GC) therapy, which reduces metabolic cell activity. In this setting, aortic/arterial wall thickening (visible on CT or MRI) is still present due to a delayed morphological vascular response [28].

There are currently no guidelines regarding PET imaging acquisition for LVV and PMR, even though standardization is of the utmost importance for facilitating clinical studies and for daily clinical practice.

The aim of this joint paper is to provide recommendations and statements, based on the available evidence in the literature and consensus of experts in the field, for patient preparation and FDG-PET/CT(A) acquisition and interpretation in the diagnosis and follow-up of patients with suspected or diagnosed LVV and/or PMR. This position paper aims to set an internationally accepted standard for FDG-PET/CT(A) imaging and reporting of LVV and PMR. An additional aim is to facilitate prospective clinical studies and pooling of future multi-center data. Other imaging modalities applied in LVV diagnostics, such as MRI angiography and ultrasound, are beyond the scope of this document.

## FDG-PET/CT(A) procedures in LVV and PMR

### Patient preparation and FDG-PET/CT(A) image acquisition

#### Patient preparation

The main goal of adequate patient preparation is to reduce physiologic tracer uptake in normal tissues (myocardium, skeletal muscle, urinary tract and brown adipose tissue) while maintaining uptake in diseased tissues and organs. Patients are instructed to fast for at least 6 h prior to FDG administration although intake of non-caloric beverages is allowed during that period [27]. In addition, strenuous physical activities should be avoided within 24 h before FDG administration. At the moment of and after administration of FDG, patients should relax in an adequately temperature-controlled room (20–22 °C [68–71.6 °F]) to minimize physiologic uptake in muscles and brown fat [29]. In some cases, FDG uptake in brown fat can be reduced by beta-blocking drugs, e.g. orally administered 20 mg propranolol 1 h before FDG injection [30]. Prior to positioning on the table, patients are asked to void urine. Patients with FUO and suspicion of cardiac involvement (e.g. endocarditis, sarcoidosis) must prepare with a special diet to reduce physiological myocardial uptake of FDG. Patient preparation for cardiac FDG-PET imaging is based on increasing the provision of fatty acids to the heart and decreasing physiological uptake of glucose by the myocardium. The SNMMI/ASNC/Society of Cardiovascular Computed Tomography (SCCT) guidelines and SNMMI/ASNC consensus document recommend preparation with a fat-enriched diet lacking carbohydrates for 12–24 h prior to the scan, a 12–18 h fast, and/or the use of intravenous unfractionated heparin approximately 15 min prior to FDG injection [31, 32].

#### Serum glucose levels before FDG administration

Previous studies have shown that FDG uptake is reduced if serum glucose levels exceed 7 mmol/L (126 mg/dL) [33–35], thereby rapidly and efficiently shunting FDG to organs with a high density of insulin receptors (e.g. skeletal and cardiac muscles), resulting in altered FDG biodistribution and suboptimal image quality [36].

The impact of glucose levels on FDG uptake in inflammatory lesions is less well investigated. A study by Rabkin et al. in 123 patients with suspected infection demonstrated that hyperglycemia at the time of study had no significant impact on the false-negative rate [33]. However, a prospective study in 195 patients evaluating the impact of fasting glucose levels on arterial uptake showed a negative correlation between uptake in the arterial wall and pre-scan glucose levels, as well as increased blood pool activity with increased glucose levels [35]. In general, efforts should be made to reduce blood glucose to the lowest possible level, but glucose levels below 7 mmol/L (126 mg/dL) are preferable.

#### Glucocorticoids and FDG administration

Glucocorticoids (GC) may reduce vascular wall uptake of FDG; the available data regarding the effect of GC withdrawal on FDG uptake are scarce. Nielsen et al. recently confirmed that diagnostic accuracy of LVV with FDG-PET remained for 3 days after initiation of GC, after which the signal decreased significantly [37–39]. Thus there may be a diagnostic window of opportunity within 3 days of initiation of GC.

A brief withdrawal of GC could “restore” pathological FDG uptake and reduce the likelihood of a false-negative result, but this is not known. At the same time, GC withdrawal may pose risks to the patient. In the case of GCA, especially if temporal artery or ocular involvement is suspected, administration of GC cannot be delayed or withdrawn due to possible ischemic complications. In other conditions such as PMR or TA, withdrawing or delaying therapy until after PET can be permitted unless there is risk of ischemic complications (Table 1).

**Table 1:** Recommendations for patient preparation and image acquisition for FDG-PET/CT in LVV and PMR

Parameter	Recommendation
Dietary preparation	Fast for at least 6 h prior to FDG administration In the case of fever of unknown origin (FUO) or suspected cardiac involvement: Consider a fat-enriched diet lacking carbohydrates for 12–24 h prior to the scan, a 12–18 h fast, and/or the use of intravenous unfractionated heparin approximately 15 min prior to FDG injection
Blood glucose levels	Preferably <7 mmol/L (126 mg/dL)
Glucocorticoids	Withdraw or delay therapy until after PET, unless there is risk of ischemic complications, as in the case of GCA with temporal artery involvement. FDG-PET within 3 days after start of GC is optional as a possible alternative [37, 39]
Patient positioning	Supine, arms next to the body
Scan range	Head down to the feet
Scan duration	3D: 2–3 min/bed position*
Dose of FDG injection	3D: 2–3 MBq/kg (0.054–0.081 mCi/kg) body weight*
Incubation time after FDG injection	Standard 60 min
PET/CT	Low-dose non-contrast CT for attenuation correction and anatomical reference

*Depending on vendor suggestion of camera system

The use of GC may also increase FDG uptake in the liver, resulting in underestimation and/or under-scoring of vascular FDG uptake [40].

#### Acquisition time after FDG administration

A minimum of 60 min between intravenous FDG administration and acquisition has been recommended for adequate tracer biodistribution [27]. Delayed acquisitions increase the vascular-to-blood pool ratio, hence increasing contrast resolution [35], and could make the measured vascular uptake more accurate [41]. However, as the majority of LVV studies have been performed at 60 min, PET-positive criteria at delayed time points have not yet been evaluated in this setting and may differ slightly from those defined at the standard time interval. In contrast to FDG-PET studies evaluating metabolic activity of atherosclerotic lesions, studies comparing early (1 h) versus delayed (3 h) imaging in LVV are scarce [42]. A small prospective study in 23 patients with suspicion of LVV concluded that delayed imaging at 3 h yielded a more detailed image of the arterial wall, mainly due to decreased blood pool activity [43]. The recently published EANM position paper on the use of FDG-PET in atherosclerosis recommends an interval of 2 h between FDG administration and acquisition [44]. Currently, there is not enough evidence to apply the same time window for LVV. At this time, we recommend an uptake interval of at least 60 min. Standardization of the time interval is essential, especially when using semiquantitative analyses and when comparing FDG uptake on follow-up studies and between institutes.

#### Patient positioning and acquisition parameters

There are currently no guidelines for image acquisition in LVV or PMR, but whole-body acquisition from head to knee (optionally including the feet) in the supine position with the arms next to the body is recommended, because (PMR) patients are generally unable to hold their arms above their head. For FDG-PET/CT imaging, a low-dose non-contrast CT must be performed for attenuation correction and anatomical localization. Alternatively, a diagnostic contrast-enhanced CT may be performed according to applicable local or national protocols and guidelines. A contrast-enhanced CTA is useful for identifying stenotic lesions in TA, but data are insufficient to support its routine use for GCA LVV [45]. When using a contrast-enhanced CTA, a low-dose CT scan should be performed prior to intravenous contrast injection for attenuation correction and subsequent standardized uptake value (SUV) calculations. The impact of intravenous contrast agents on the accuracy of attenuation correction is considered acceptable only when CT data are collected in the equilibrium or venous phase (i.e. delayed acquisition), with the advantage of radiation dose reduction [26]. Detection of smaller vascular structures in the head and neck region can be improved by increasing the acquisition time (~ doubled) per bed position to improve image quality, and applying larger image matrices (thus smaller voxels) [46]. This will reduce the partial volume effect of smaller structures, provided appropriate high-resolution image reconstruction settings are chosen, e.g. minimal image filtering during reconstruction and appropriate number of iterations/subsets to ensure sufficient convergence and/or contrast recovery by the iterative reconstruction process. When available, time-of-flight information should be used during reconstruction.

**Table:** 

Consensus recommendations (see supplement 1)
• Recommend patient fasting for at least 6 h prior to FDG administration, although intake of non-caloric beverages is allowed during that period (evidence level II, grade B).
• Normal blood glucose levels are desirable, but glucose levels below 7 mmol/L (126 mg/dL) are preferable (evidence level II, grade B).
• Withdraw or delay GC therapy until after PET, unless there is risk of ischemic complications, as in the case of GCA with temporal artery involvement. FDG-PET within 3 days after start of GC is optional as a possible alternative (evidence level III, grade B).
• A minimum interval of 60 min is recommended between FDG administration and acquisition for adequate biodistribution (evidence level III, grade B).

### Interpretation and reporting of FDG-PET/CT(A)

#### Interpretation criteria

**Fig. 1:**
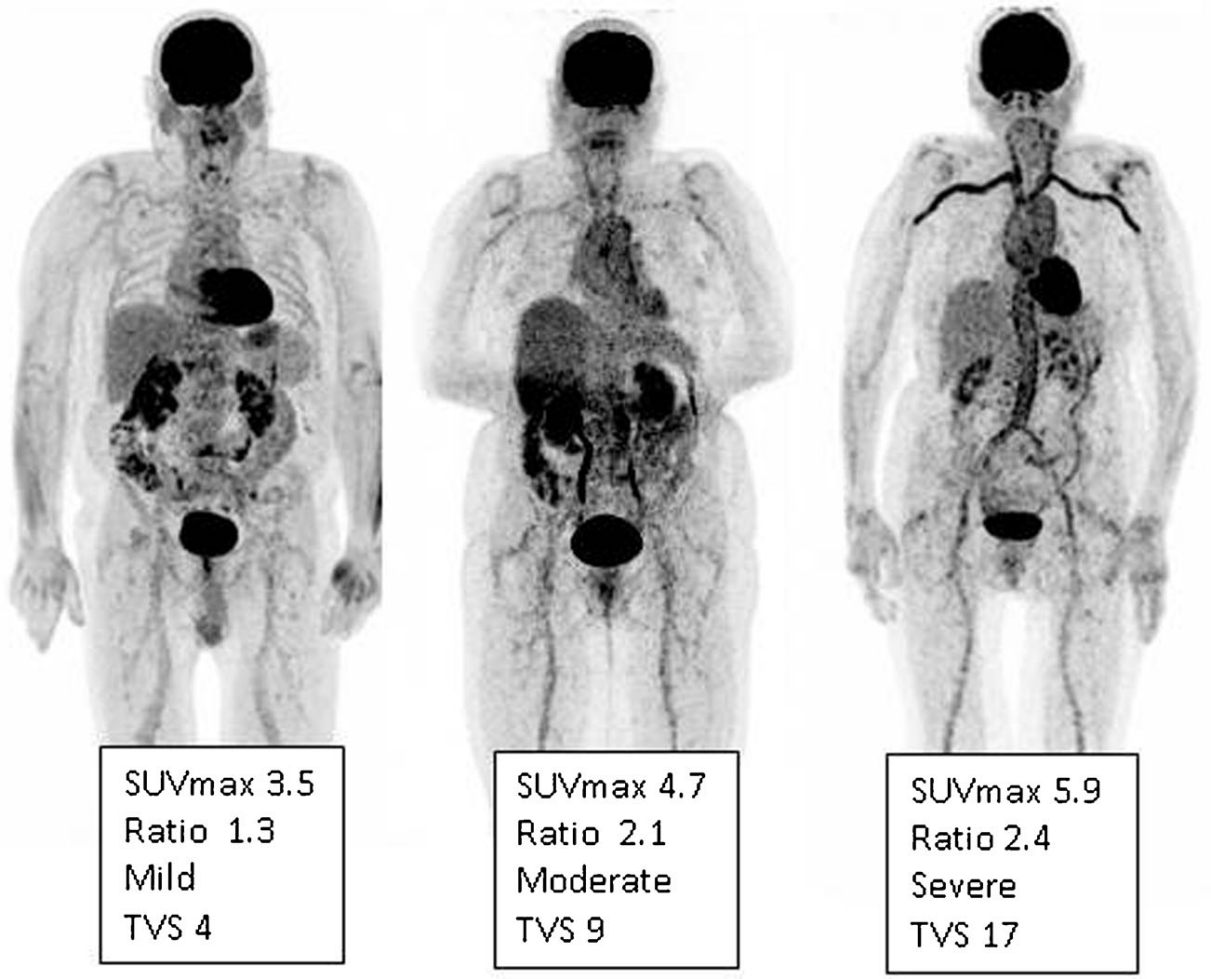
FDG-PET. Low (grade 1), intermediate (grade 2), and high (grade 3) LVV FDG uptake patterns including SUV_max_ values of the thoracic aorta in patients with GCA. Ratio is defined as average SUV_max_ of the thoracic aorta divided by the liver region. The total vascular score (TVS) is the highest for the right-positioned patient

Several factors may significantly influence the arterial wall FDG uptake, and must be taken into consideration for interpretation of FDG-PET in LVV and PMR. For clinical routine, interpretation criteria must be uniform, reproducible, and easy to use. Many PET interpretation criteria have been proposed (Table 2), and evidence from the last 15 years supports the use of a visual grading scale (vascular to liver uptake) (Fig. 1). We propose the use of a standardized 0-to-3 grading system as follows: 0 = no uptake (≤ mediastinum); 1 = low-grade uptake (< liver); 2 = intermediate-grade uptake (= liver), 3 = high-grade uptake (> liver), with grade 2 possibly indicative and grade 3 considered positive for active LVV (Table 3) [25, 73]. A total vascular score (TVS) can be determined, for instance, at seven different vascular regions (thoracic aorta, abdominal aorta, subclavian arteries, axillary arteries, carotid arteries, iliac arteries, and femoral arteries) as negative (0) or positive, further scored semiquantitatively as 1 (minimal but not negligible FDG uptake), 2 (clearly increased FDG uptake), or 3 (very marked FDG uptake). Therefore, a TVS could be calculated ranging from 0 (no vascular FDG uptake in any of the seven vascular regions) to 21 (vascular FDG uptake scored 3 in all seven territories).

**Table 2:** Literature review of the FDG-PET interpretation criteria used in LVV

	PET evaluation criteria	References
	Visual analysis	
Giant cell arteritis / PMR	Uptake pattern	[7, 47]
	Grading	[19, 21, 48–58]
	Total vascular score	[59, 60]
	Semiquantitative	
	SUV	[38, 48, 53]
	Target-to-liver ratio	[61]
	Target-to-lung ratio	[12]
	Target-to-blood pool	[62]
Takayasu arteritis	Visual analysis	
	Grading	[48, 51, 52, 57, 58, 63–69]
	Semiquantitative	
	SUV	[70–72]
	Target-to-blood pool	[70]

**Table 3:** Proposed standardized FDG-PET/CT(A) interpretation criteria in LVV

	Recommended PET interpretation criteria
For clinical use	LVV visual grading (GCA and TA) Grade 0: No vascular uptake (≤ mediastinum) Grade 1: Vascular uptake < liver uptake Grade 2: Vascular uptake = liver uptake, may be PET-positive Grade 3: Vascular uptake > liver uptake, considered PET-positive
	PMR associated visual assessment (only GCA) Grade 0: No uptake Grade 1: Uptake < liver uptake Grade 2: Uptake = liver uptake Grade 3: Uptake > liver uptake Increased metabolic activity of the scapular and pelvic girdles Increased metabolic activity of the knee bursae and capsule Increased metabolic activity at the site of the cervical and lumbar interspinous bursae Increased metabolic activity of the trochanteric and ischial bursae
In general for research only	PET semiquantitative analysis* Target: Average SUV_max_ artery of the vascular ROIs Blood pool: Average SUV_mean_ of several vein ROIs TBR = average SUV_max_ artery / average SUV_mean_ vein Liver: SUV_max_ of a liver region, preferably the right lobe TBR = average SUV_max_ artery / SUV_max_ of a liver region Vascular targets: - Carotid arteries - Subclavia arteries - Axillary arteries - Vertebral arteries - Ascending aorta - Aortic arch - Pulmonary arteries - Descending aorta - Abdominal aorta Joints: scapulae and pelvic girdles, knees, cervical and lumbar interspinous bursae, trochanteric and ischial bursae
For clinical use	Contrast-enhanced (PET/)CTA Regular vascular wall thickness (mm) Contrast enhancement Presence of stenosis / aneurysm

*TBR* target-to-background ratio; *SUV* standardized uptake value; *ROI* region of interest; *TA* Takayasu arteritis; *PMR* polymyalgia rheumatica; *GCA* giant cell arteritis.

*SUV using EARL criteria [26]

**Fig. 2:**
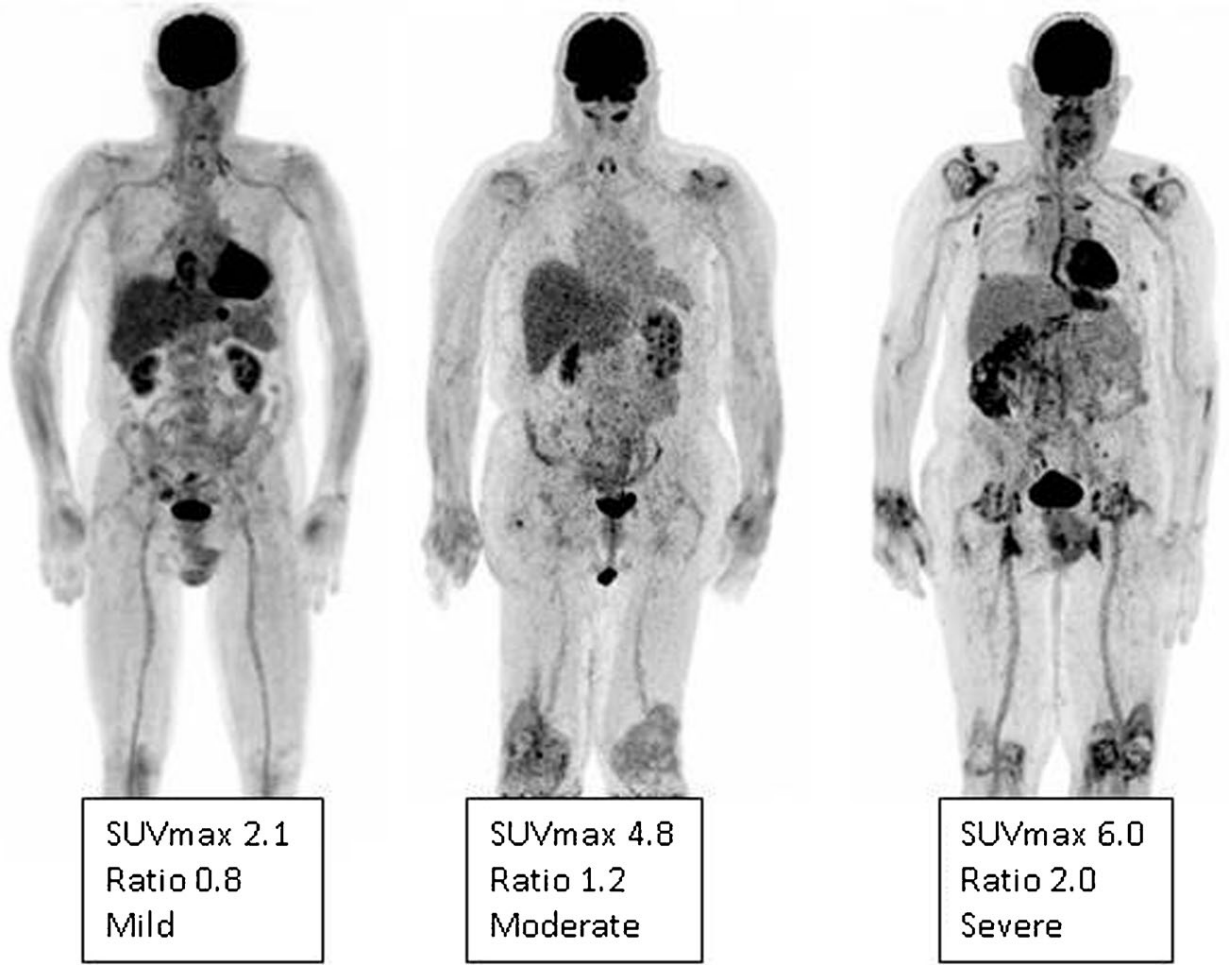
FDG-PET. Low (grade 1), intermediate (grade 2), and high (grade 3) FDG uptake patterns of the large joint regions in PMR patients, including SUV_max_ of the shoulders. Ratio is defined as average SUV_max_ in the shoulders divided by the liver region. The total number and intensity of affected joints is the highest for the right-positioned patient

As PMR and GCA frequently overlap, typical FDG joint uptake patterns should be reported, including uptake in glenohumeral synovia, subacromial-subdeltoid bursa, supraspinatus tendinitis and biceps synovitis (shoulder), trochanteric/ischial bursa, hip synovia, interspinous regions of the cervical and lumbar vertebrae, or the synovial tissue of the knees if present, including the use of a standardized 0-to-3 grading system [74, 75] (Fig. 2).

Atherosclerotic vascular uptake [76, 77], frequent with aging, may be a source of false positivity for LVV evaluation, despite a classical “patchy” uptake pattern. Uptake in iliofemoral arteries should be interpreted with caution, because this is a frequent site of atherosclerosis. Taking these considerations into account, vascular inflammation in LVV on FDG-PET classically appears as a smooth linear pattern, involving the aorta and its main branches (subclavian, carotid or vertebral arteries, pulmonary arteries specifically in TA), but not all main branches have to be involved.

##### Quantification issues requiring further clarification

Several semiquantitative methods have also been proposed, from simple SUV metrics to target-to-background ratios (TBR) (Table 2). The clinical utility of SUV or TBR for initial diagnosis of LVV or PMR is currently unknown, and their use is not recommended. However, their relevance for recurrence or follow-up evaluation may be of interest. Simple SUV metrics do not appear relevant in initial diagnosis, due to the high overlap between patients and controls [62] and the potential loss of specificity [48]. TBR methods using lung [12], liver [61], or blood pool [62, 70] as a reference have been proposed, mainly in GCA studies. A target-to-blood pool method was recently applied successfully in LVV, was highly reproducible in atheroma [78], and is currently recommended by the cardiovascular committee of the EANM for the assessment of vascular wall inflammation in this setting [44]. Based on the few promising results in LVV [40, 62], we encourage the use of this target-to-blood pool method in LVV for research studies. The use of TBR instead of SUV is recommended, as the use of a ratio between two measurements limits the effects on signal quantification of errors in patient weight, injected radiotracer dose, and imaging time point [44].

The normalization of the arterial wall uptake to the background activity of venous blood pool provides a good reference for assessing vascular inflammation [40]. Also, grading of arterial inflammation against the liver background is an established method [25, 40].

Regions of interest (ROIs) are drawn around the majority of the target arterial structure, while the chance of including surrounding FDG uptake within the ROI needs to be minimized [40]. For background quantification, the ROI is projected on the right lobe of the liver to reduce the chance of including the various veins and arteries running through the liver. For blood pool, an ROI is drawn centrally in the blood pool of the (inferior or superior) caval vein.

TBR varies as a function of blood pool activity, which can be affected by many factors, including (1) FDG uptake in circulating blood cells, (2) chronic renal insufficiency, and (3) blood glucose levels [79, 80]. A study by Lensen et al. in patients with atherosclerosis showed that results were affected by several data acquisition parameters, i.e. FDG uptake time and SUV normalization [81]. Although the individual factors may not have a large impact by themselves, the cumulative effects of these factors may result in substantial differences in reported SUVs throughout studies and within multi-center trials. Repeated PET/CT examinations should be performed using the same protocol as in the previous studies. Semiquantitative analysis should be similarly performed (in order to compare PET/CT results). For treatment response evaluation, it is important to have basic (prior to therapy) PET/CT results, as the detection of even slight FDG uptake in the region of the initial lesion should be considered a residual inflammatory process.

**Table:** 

Consensus recommendations
• We propose the use of a standardized grading system: 0â€‰=â€‰no uptake (â‰¤ mediastinum); 1â€‰=â€‰low-grade uptake (< liver); 2â€‰=â€‰intermediate-grade uptake (= liver), 3â€‰=â€‰high-grade uptake (> liver), with grade 2 considered possibly positive and grade 3 positive for active LVV (evidence level II, grade B).
• Typical FDG joint uptake patterns including scapular and pelvic girdles, interspinous regions of the cervical and lumbar vertebrae, or the knees should be evaluated and reported if present (evidence level II, grade B).
• Normalization of the arterial wall uptake to the background activity of venous blood pool provides a good reference for assessing vascular inflammation (evidence level II, grade B).
• Grading of arterial inflammation against the liver background is an established method (evidence level II, grade B).

### Diagnostic accuracy of FDG-PET/CT(A) for LVV and PMR

In general, the diagnostic performance of FDG-PET for the detection of LVV is good; individual studies are summarized in Table 4, and meta-analyses are summarized in Table 5. A recent meta-analysis of eight studies including 170 LVV patients with GCA or TA and 230 controls confirmed that FDG-PET offers good diagnostic performance for the identification of LVV [86]. The diagnostic performance of FDG-PET was higher for the detection of GCA than TA (87% vs. 58%, respectively; *p* < 0.0001) [73, 86], but was impaired in patients under GC and/or immunosuppressive treatment at the time of imaging [73]. Of note, patients with TA are more often receiving long-term treatment at the time of imaging than patients with GCA. For the diagnosis of patients with GCA, FDG-PET demonstrated high pooled sensitivity (90%) and specificity (98%), without significant heterogeneity, in a meta-analysis of four pooled studies including 57 patients with giant cell arteritis and 176 controls [73]. These findings are in line with a previous meta-analysis including GCA patients evaluated by FDG-PET, showing pooled sensitivity and specificity of 80% and 89%, respectively [87]. In TA, FDG-PET demonstrated pooled sensitivity of 87% and specificity of 73% for the assessment of disease activity in a recent meta-analysis of seven studies including 191 patients with TA, with significant heterogeneity [73]. These findings are in line with a previous meta-analysis including TA patients evaluated by FDG-PET, showing pooled sensitivity and specificity of 70% and 77%, respectively [88]. The specificity of FDG-PET increased to 84% when considering studies using National Institutes of Health (NIH) criteria [89] as the disease activity assessment scale [73]. Visual analysis showed that high FDG uptake correlated well with the presence of markers of disease activity in TA, but vascular uptake was observed in up to 67% of TA patients without markers of activity [73].

**Table 4:** Systematic review of main findings of individual studies assessing the diagnostic accuracy of FDG-PET or FDG-PET/CT(A) at baseline in patients with large vessel vasculitis and/or PMR

LVV type (indication)	Study type	Cases	Controls	IS therapy before baseline PET	Diagnostic criteria used for LVV	FDG injected activity	Time between FDG injection and PET acquisition (min)	Glucose serum levels before PET (mg/dL [mmol/L])	PET analysis	Threshold used for diagnosis of LVV at PET	Sensitivity	Specificity	Authors	Year
GCA and PMR (diagnosis)	P	15	9	33%	ACR, clinical criteria or TAB	4 MBq/kg (0.11 mCi/kg)	90	NR	QA (visual) and SQA (vessel wall SUV_max_/blood pool SUV_mean_)	QA: high vascular uptake	66.7% (QA)	100% (QA)	Lariviere et al. [82]	2016
GCA (diagnosis)	R	18	53	33%	ACR, clinical criteria or TAB	3 MBq/kg (0.081 mCi/kg)	60 ± 5	NR	QA (visual) and SQA (aortic SUV_max_ and aortic/liver, aortic/superior cava, aortic/inferior cava SUV_max_ ratios)	QA1: first impression QA2: diffuse vascular uptake = liver uptake QA3: diffuse vascular uptake > liver uptake	56% (QA1)100% (QA2)83% (QA3))	98% (QA1)51% (QA2)91% (QA3)	Stellingwerff et al. [40]	2015
GCA + PMR (diagnosis)	R	25	6	12%	ACR (GCA), Healey (PMR), clinical, biochemical criteria or TAB	3 MBq/kg (0.081 mCi/kg)	60 ± 5	NR	QA (visual)	QA1: first impression QA2: diffuse vascular uptake = liver QA3: diffuse vascular uptake > liver QA4: diffuse vascular uptake > femoral artery	92% (QA1)100% (QA2)100% (QA3)80% (QA4)	90% (QA1)60% (QA2)98% (QA3)96% (QA4)	Lensen et al. [25]	2015
GCA (diagnosis)	P	32	20	53%	TAB	370 MBq (10 mCi)	60	NR	SQA (vessel SUV_max_)	SQA: vessel SUV_max_ cutoff 1.89	80% (SQA)	79% (SQA)	Prieto-Gonzalez et al. [38]	2014
GCA (diagnosis)	R	11	11	73%	TAB	4 MBq/kg (0.11 mCi/kg)	60	< 180 (10)	SQA (aortic/liver, lung, or venous blood pool SUV_max_ ratio)	SQA: aortic/venous blood pool SUV_max_ ratio cutoff 1.53	81.8%(SQA)	91% (SQA)	Besson et al. [62]	2014
PMR	R	14	17	0	Chuang and Healey	370 MBq (10 mCi)	60	NR	QA (visual) and SQA (vessel SUV_max_)	QA: mild vascular uptake (< liver uptake)	64.3% (QA)	76.5% (QA)	Yamashita et al. [83]	2012
GCA (diagnosis)	P	23	36	0	ACR, TAB or duplex sonography	361 ± 54 MBq (9.76 ± 1.5 mCi)	60	NR	SQA (vessel/liver SUV_max_)	SQA: vessel/liver SUV ratio cutoff 1	88.9% (SQA)	95.1% (SQA)	Hautzel et al. [61]	2008
PMR (diagnosis)	P	13	6	0	Chuang and Healey	450 MBq (12.2 mCi)	90	NR	QA (visual) and SQA (vessel/lung uptake ratio)	NR	92.3% (QA)	100% (QA)	Moosig et al. [12]	2004
GCA + PMR (diagnosis)	P	25	44	0	TAB and ACR (GCA) or Hunder and Healey (PMR)	6.5 MBq/kg (0.18 mCi/kg)	60	NR	QA (visual)	QA: moderate uptake (= liver uptake)	76% (QA)	77% (QA)	Blockmans et al. [7]	2000
TA (diagnosis and disease activity)	R	51	50	75%	ACR and NIH	370 MBq (10 mCi)	60	< 150 (8.5)	QA (visual) and SQA (vessel SUV_max_ and vessel SUV_max_/liver SUV_mean_)	QA: intense uptake (> liver uptake) in the ascending aorta, moderate uptake (= liver uptake) in the aortic arch and large aortic branch, and mild uptake (< liver uptake) in the descending or abdominal aorta	83.3% (QA)	90% (QA)	Santhosh et al. [68]	2014
TA (disease activity)	CS	22	NR	77%	ACR, NIH, DEI-Tak, clinical and biochemical criteria	480 MBq (13 mCi)	60	NR	QA (visual) and SQA (vessel SUV_max_ and vessel SUV_max_/liver SUV_mean_)	QA: moderate uptake (= liver uptake) for aorta and mild uptake for other vessels	100% (QA)	88.9% (QA)	Karapolat et al. [65]	2013
TA (disease activity)	R	39	40	74%	ACR, JCS, and NIH	3.7 MBq/kg (0.1 mCi/kg)	69	< 120 (7)	QA (visual) and SQA (vessel SUV_max_ and vessel SUV_max_/inferior cava SUV_mean_)	SQA: vessel SUV_max_ cutoff 2.1	92.6% (SQA)	91.7% (SQA)	Tezuka et al. [70]	2012
TA (disease activity)	R	38	NR	37%	ACR and NIH	370 MBq (10 mCi)	40–60	74–122 (4–7)	QA (visual) and SQA (vessel/liver SUV_max_)	QA: moderate vascular uptake (= liver uptake)	75% (QA)	64.3% (QA)	Lee et al. [66]	2012
TA (disease activity)	R	28	NR	70%	ACR and NIH	5 MBq/kg (0.135 mCi/kg)	60	NR	QA (visual) and SQA (vessel SUV_max_ and vessel SUV_max_/liver SUV_mean_)	QA: moderate vascular uptake (= liver uptake)	69.2% (QA)	33.3% (QA)	Arnaud et al. [64]	2009
TA (disease activity)	R	32	NR	31%	ACR and NIH	551 ± 55 MBq (15 ± 1.5 mCi)	60	97 ± 16 (5.5 ± 1)	QA (visual)	QA: moderate uptake (= liver uptake) for aorta and mild uptake for other vessels	78% (QA)	87% (QA)	Lee et al. [67]	2009
TA (disease activity)	P	14	6	79%	ACR	6 MBq/kg (0.16 mCi/kg)	45	NR	SQA (vessel SUV_max_)	SQA: SUV_max_ cutoff 1.3	90.9% (SQA)	88.8% (SQA)	Kobayashi et al. [72]	2005
TA (disease activity)	R	18	NR	61%	ACR and angiography	185–259 MBq (5-mCi)	90	NR	QA (visual)	QA: mild vascular uptake (< liver uptake)	92% (QA)	100% (QA)	Webb et al. [69]	2004
GCA, PMR and TA (diagnosis and disease activity)	R	25	15	0 (at baseline)	NR	199–478 MBq (5.4–12.9 mCi)	50–60	NR	QA (visual) and SQA (vascular SUV_mean_)	QA: summed vascular visual score cutoff 8SQA: average vascular SUV_mean_ cutoff 0.697	84% (QA)96% (SQA)	86.7% (QA)86.7% (SQA)	Castellani et al. [84]	2016
GCA + TA (diagnosis)	P	43	15	NR	Clinical, biochemical criteria or TAB	7 MBq/kg (0.19 mCi/kg)	180	102.2 ± 24(5.6 ± 1)	SQA1 (aortic SUV_max_)SQA2 (aortic wall SUV_max_/lm SUV_max_)	SQA1: aortic SUV_max_ cutoff 1.74SQA2: aortic wall SUV_max_/lm SUV_max_ cutoff 1.34	80% (SQA1)100% (SQA2)	83.3% (SQA1)94% (SQA2)	Martínez-Rodríguez et al. [43]	2014
GCA + TA + other vasculitis (diagnosis)	R	31	33	50%	ACR, clinical and biochemical criteria	3.7 MBq/kg (0.1 mCi/kg)	60 ± 10	< 140 (7.8)	QA (visual) and SQA (vessel SUV_max_) or JA (QA and radiological/clinical elements)	QA1: mild vascular uptake (< liver uptake)QA2: moderate vascular uptake (= liver uptake)SQA: vessel SUV_max_ cutoff 2.4	93.5% (QA1)64.5% (QA2)74.2% (SQA)93.5% (JA)	75.7% (QA1)84.8% (QA2)78.8% (SQA)93.9% (JA)	Rozzanigo et al. [85]	2013
GCA + TA (diagnosis)	P	30	31	51%	ACR, clinical and biochemical criteria	5 Mbq/kg (0.29 mCi/kg)	45	< 180 (10)	QA (visual)	QA: moderate uptake (= liver uptake) for aorta and mild uptake for other vessels	73.3% (QA)	83.9% (QA)	Fuchs et al. [52]	2012
GCA + TA (diagnosis)	R	24	18	79%	Clinical and biochemical criteria or TAB	5 MBq/kg (0.135 mCi/kg	60	104 ± 25 (5.8 ± 1.6	QA (visual)	QA: moderate vascular uptake (= liver uptake)	92% (QA)	91% (QA)	Förster et al. [21]	2011
GCA + TA (diagnosis)	R	20	20	40%	ACR or TAB	350–400 MBq (9.5–10.8 mCi)	60	NR	QA (visual) and SQA (vessel SUV_max_)	QA: intense vascular uptake (> liver uptake)SQA: SUV_max_ cutoff 2.24	65% (QA)90% (SQA)	80% (QA)45% (SQA)	Lehmann et al. [48]	2011
GCA and TA (diagnosis and disease activity)	P	13	8	62%	ACR and BVAS, duplex sonography, MRI or TAB	390–488 MBq (10.5–13.2 mCi)	60	< 120 (6.7)	QA (visual) and SQA (vessel SUV_max_)	NR	92.3% (QA)	100% (QA)	Henes et al. [53]	2008

Abbreviations: GCA = giant cell arteritis;TA = Takayasu arteritis;LVV = large vessel vasculitis;DOR = diagnostic odd ratio;AUC = area under the curve;N/A = not available

IS = immunosuppressive; NR = not reported. Study type: P = prospective; R = retrospective; CS = cross sectional. Type of vasculitis: LVV = large vessel vasculitis; PMR = polymyalgia rheumatica; RF = retroperitoneal fibrosis. Diagnostic criteria: ACR = American College of Rheumatology; NIH = National Institutes of Health; TAB = temporal artery biopsy; MRI = magnetic resonance imaging; JCS = Japanese Circulation Society; BVAS = Birmingham Vasculitis Activity Score; DEI-Tak = Disease Extent Index—Takayasu. PET analysis: QA = qualitative analysis; SQA = semiquantitative analysis; JA = joint analysis; SUV_max_ = maximum standardized uptake value; SUV_mean_ = mean standardized uptake value

**Table 5:** Main findings of available meta-analyses on the diagnostic accuracy of FDG-PET or FDG-PET/CT(A) in patients with large vessel vasculitis

LVV	Studies included	Number of patients	Sensitivity (95% CI)	Specificity (95% CI)	Positive likelihood ratio	Negative likelihood ratio	DOR	AUC	Authors	Year
GCA	3	66	83.3%(72–91)	89.6%(80–96)	7.10(2.91–17.36)	0.2(0.11–0.34)	37.93(11.55–124.5)	0.88	Lee et al. [86]	2016
	4	57	90%(79–96)	98%(94–99)	28.7(11.5–71.6)	0.15(0.07–0.29)	256.3(70.8–927)	0.98	Soussan et al. [73]	2015
	6	101	80%(63–91)	89%(78–94)	6.73(3.55–12.77)	0.25(0.13–0.46)	N/A	0.84	Besson et al. [87]	2011
TA	7	191	87%(78–93)	73%(63–81)	4.2(1.5–12))	0.2(0.1–0.5)	19.8(4.5–87.6)	0.91	Soussan et al. [73]	2015
	6	76	70.1%(58.6–80)	77.2%(64.2–87.3)	2.31(1.11–4.83)	0.34(0.14–0.82)	7.5(1.65–34.07)	0.805	Cheng et al. [88]	2013
LVV (GCA and TA)	8	170	75.9%(68.7–82.1)	93%(88.9–96)	7.27(3.71–14.24)	0.3(0.23–0.4)	32.04(13.08–78.45)	0.86	Lee et al. [86]	2016

Abbreviations, see Table 4

**Table 6:** Recommendations for patient preparation and image acquisition for the CTA scan

Patient positioning	Supine, arms next to the body for hybrid PET/CTA; otherwise, arms should be elevated.
Scan volume	Entire aorta including the cervical, upper extremity, visceral and renal, pelvic, and proximal lower extremity arterial branches
Contrast material administration	80 to 150 mL iodinated low-osmolar or iso-osmolar contrast material with concentrations of 300 to 400 mg iodine per mL is injected at flow rates of 3.0–5.0 mL/s via antecubital vein.
Specific CTA settings	Optimal arterial contrast phase: Bolus tracking or test bolus technique, scanning in cranio-caudal direction Avoidance of aortic motion artifacts: ECG triggering
Specific CT machine settings	Refer to individual CT scanner recommendations, as parameters and protocols may differ among vendors and machines.

The precise evaluation of diagnostic accuracy of FDG-PET for the diagnosis of LVV faces several hurdles. First, in some patients, FDG-PET represents the only modality that allows for the diagnosis of LVV, and therefore cannot be compared to a gold standard. For GCA, the diagnosis is usually classified according to the American College of Rheumatology (ACR) criteria [90], which include cranial symptoms, the presence of an elevated erythrocyte sedimentation rate (ESR), and a positive superficial temporal artery biopsy (TAB). Arterial wall inflammation in GCA, however, is characterized by a segmental distribution, and can be absent in the excised segment of the superficial temporal artery. The presence of aortitis in patients with PMR is even more difficult to confirm, as FDG uptake is most often the only modality that allows for the detection of inflammatory activity in large vessels. The diagnosis of TA is usually based on the NIH score [89], which integrates clinical, biological, and radiological criteria. Several studies, however, have found that there might be discrepancies between the activity of TA evaluated with the NIH score and the results of FDG-PET imaging [73]. This raises the question of whether FDG-PET is more sensitive than the NIH score for detecting and assessing TA, or whether this vascular signal has no relation with active progressive disease. Second, patients with suspected GCA often immediately receive high-dose GC before imaging, which has an impact on the intensity of arterial FDG uptake subsequently measured with PET. The accuracy of FDG-PET can therefore vary in relation to the delay between the initiation of GC therapy and imaging. Third, the accuracy of a diagnostic test is influenced by the criteria used to define the presence of the disease. To date, there are no definitive consensus criteria for the presence of vascular inflammation with FDG-PET in LVV or PMR. In summary, based on the available evidence, FDG-PET imaging has high diagnostic value for the detection of LVV or PMR. Future studies are needed to select the most clinically relevant and reproducible criteria for defining the presence of LVV with FDG-PET, as well as to test the clinical impact of FDG-PET imaging on the management of patients with suspected LVV.

**Table:** 

Consensus statement
• Based on the available evidence, FDG-PET imaging exhibits high diagnostic performance for the detection of LVV and PMR (evidence level II, grade B).
• Further studies are needed to select the most clinically relevant and reproducible criteria for defining the presence of LVV with FDG-PET, as well as to test the clinical impact of FDG-PET imaging on the management of patients with suspected LVV.

### CT angiography in LVV and PMR

#### Image acquisition

**Fig. 3:**
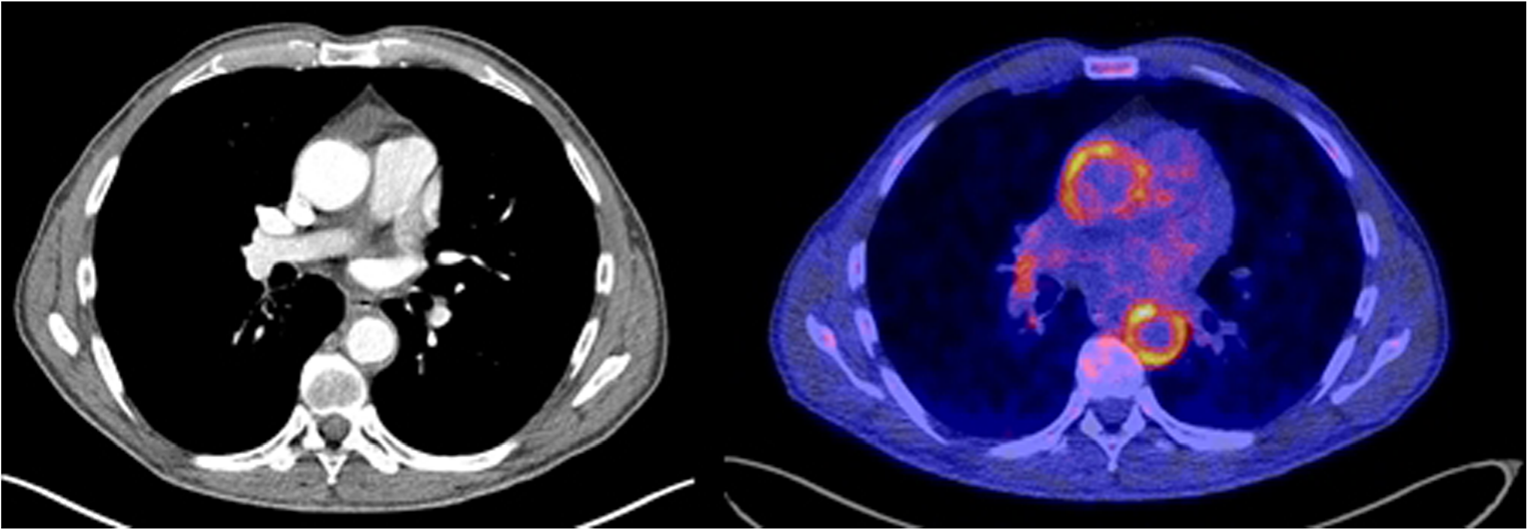
FDG-PET/CTA. On the left, a transaxial view of a contrast chest CT in a 67-year-old man with GCA, with an enlarged diameter of the ascending aorta of 41 × 41 mm and moderately increased wall thickness of 3.1 mm, and severely increased wall thickness of 4.7 mm of the descending aorta (diameter of 30 × 31 mm). On the right, the fused transaxial images of the contrast chest CT and FDG-PET showing highly elevated FDG uptake (average SUV_max_ 5.5) in the ascending and descending aorta

Little data has been published concerning the additional value of CTA for the diagnosis of LVV. Such evaluation could be of interest by providing morphological information on the vasculature in a “one-stop shop” procedure when using hybrid PET/CTA imaging (Fig. 3). In acute disease stages, CTA can focus on the vascular lumen for both the detection and characterization of stenosis and the assessment of acute complications of a critical stenosis. In chronic disease stages, CTA is an alternative to MRI for detecting late complications such as aneurysm formation and is helpful in planning percutaneous and surgical treatment. However, given the currently limited evidence supporting the use of contrast-enhanced PET/CT in LVV, further studies are necessary to assess its potential incremental value.

CTA scanning parameters should be adapted to the specific capabilities of the local scanner specifications (Table 6). In 2014, the Task Force for the Diagnosis and Treatment of Aortic Diseases of the European Society of Cardiology (ESC) produced guidelines for CTA in the diagnosis of aortic disease in adults [91]. In 2016, the American College of Radiology (ACR), the North American Society for Cardiovascular Imaging (NASCI), the Society of Interventional Radiology (SIR), and the Society for Pediatric Radiology (SPR) jointly revised their guidelines for performing body CTA [92]. These guidelines provide general information regarding CTA scanning and image post-processing, as specific scanning parameters and image reconstruction settings differ substantially among CT vendors and machines.

Generally, these guidelines recommend, if available, the use of a multi-detector-row CT (MSCT) scanner with wide z-axis or volume coverage. The scans should be performed with ECG triggering to avoid motion or pulsation artifacts of the ascending aorta [93].

Contrast material is administered through a venous catheter using an automated contrast material injector. The contrast material dose depends on the patient's body weight, body mass index, and kidney function (recent estimated glomerular filtration rate) [26].

CTA images should be reconstructed in thin slices (e.g. 1 mm thick) to allow for additional multiplanar reformation (MPR) and 3D image post-processing. Preferably, isotropic voxels should be achieved. Both filtered back-projection and iterative reconstruction algorithms can be used, with the latter providing improved image quality due to noise removal, and dose-saving potential [94]. Medium-sharp or vascular reconstruction kernels can be recommended with a reconstruction matrix of 512 × 512 pixels and angiographic window setting using a level of 100 Hounsfield units (HU) and width of 700 HU.

#### Interpretation and reporting

**Fig. 4:**
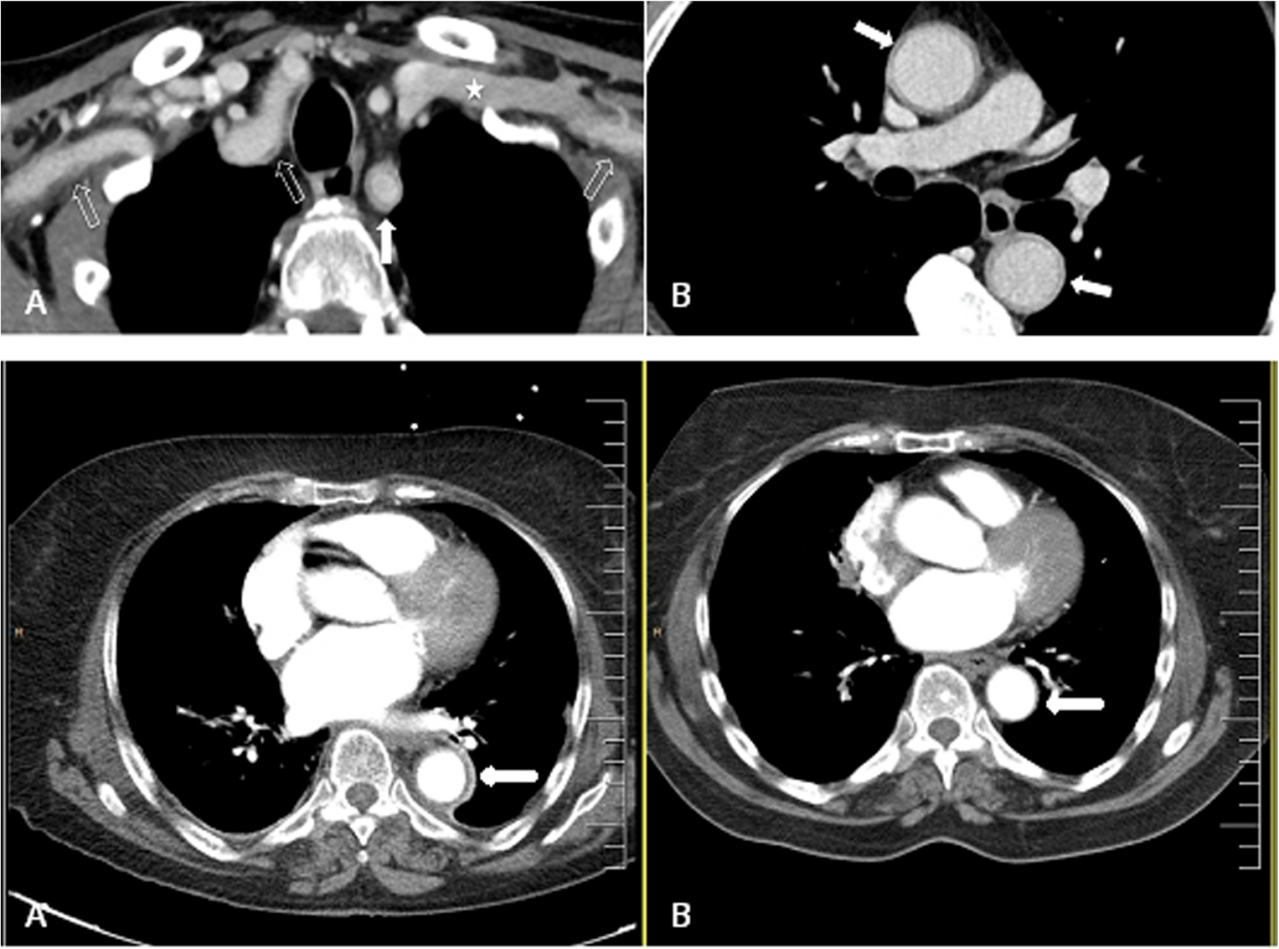
CT angiography of the chest in two patients with GCA. *Upper row* CTA of the aorta and the supra-aortic arteries in a 64-year-old male patient with giant cell arteritis. Mural thickening and contrast enhancement of the aortic wall (arrows in B). Please note hypodense inner ring delineating luminal contrast-enhanced blood from contrast-enhancing thickened aortic wall. Mural inflammatory changes are present in both subclavian arteries as visualized in cross section (bold arrow in A) and in a longitudinal section (light arrows in A). Asterisk in A indicates the left subclavian vein. *Lower row* Axial view of a CT angiography of a 76-year-old woman with GCA showing severely increased wall thickness of 5.2 mm and contrast enhancement of the descending aorta (bold arrow) (A). Contrast CT of the same patient performed 4 years earlier, with no significant aortic wall thickening (B)

According to American College of Radiology guidelines, CTA is indicated to “diagnose and localize diseases with primary manifestations in the arterial wall, including vasculitis, infection, and degenerative disorders” [92]. Arterial vessel wall thickening is the typical sign of vascular inflammation on contrast-enhanced CT images (Fig. 4). In vasculitis, mural thickening usually involves the complete circumference of the vessel wall, whereas in atherosclerosis, plaque formation starts from a focal point rather than circumferentially. CTA-based diagnosis is greatly facilitated in the absence of atherosclerotic plaques and when the thickening is not concentric. A circumferential aortic wall thickness of more than 2–3 mm with adventitial and peri-adventitial contrast enhancement is suggestive of aortitis [95, 96]. It is assumed that the degree of mural contrast enhancement is associated with the inflammatory activity, as studies have shown that aortic wall contrast enhancement can resolve during GC therapy, while the wall thickening may persist [39].

#### Diagnostic accuracy of CTA

Although CTA itself is helpful for diagnosing LVV, the diagnostic accuracy of combined FDG-PET/CTA scans remains undefined. While the inflammatory activity within the vessel wall is displayed with high sensitivity on FDG-PET images, combining FDG-PET with CTA enhances specificity by providing high-resolution anatomical details.

CTA also helps to differentiate different pathologic FDG-PET findings, as both vasculitis and atherosclerosis can demonstrate increased FDG wall uptake. Equally important in the contribution of CTA to the diagnostic accuracy of combined FDG-PET/CTA is its role in detecting structural changes and potential complications of vasculitis [96]. During the primary diagnostic work-up, CTA often helps to identify or exclude acute or symptomatic manifestations that require immediate therapy. For example, inflammatory vascular stenosis can lead to serious sequelae such as brain infarction or mesenteric ischemia with bowel necrosis. Furthermore, initial CTA may provide information on current disease stage, distribution, and duration. During disease follow-up, CTA plays a particular role in the detection and monitoring of complications such as aortic aneurysm and dissection. While stenosis is frequently observed in TA, GCA may lead to aortic or arterial dilatation. These dilated arteries may enlarge to aneurysms during the disease course, even though inflammatory activity is absent or sufficiently suppressed.

**Table:** 

Consensus recommendation
• CTA and FDG-PET have complementary roles in the diagnosis of LVV (evidence level III, grade B).
• CTA has incremental value in detecting structural vascular changes and potential complications of vasculitis (evidence level II, grade A).

### Monitoring the efficacy of immunosuppressive therapy with FDG-PET/CT(A)

For monitoring of LVV activity during and after treatment, related biomarker measurements would be helpful. Unfortunately, both the cranial GCA and the extracranial large vessel type of GCA or TA lack disease-specific serum biomarkers.

Although FDG-PET/CT(A) has proven to be an important imaging modality for diagnosis of non-temporal GCA, very limited data are available on the role of FDG-PET/CT(A) for patient management once treatment has started. With regard to the utility of FDG-PET for assessing changes in arterial wall inflammation in response to GC and methotrexate, the results are mixed, and represent only small patient cohorts.

In the only prospective study, which was conducted by Blockmans et al. (Table 7), whole-body FDG-PET/CT images were acquired at baseline and after 3 and 6 months of GC therapy [59]. The total vascular score (TVS) decreased from a mean (± SD) of 7.9 ± 5.5 at baseline to 2.4 ± 3.5 on repeat PET scan at 3 months (*p* < 0.0005), but no further decrease was seen at 6 months. In patients who experienced a relapse (recurrent signs and symptoms, together with an increase in acute-phase reactants), FDG-PET was performed within 5 days. The authors found no difference in the predictive value of FDG uptake between relapsing and non-relapsing patients.

**Table 7:** Literature review of studies using FDG-PET/CT(A) for monitoring of patients with LVV/PMR

LVV type (indication)	Study type	Cases	Controls	Therapy	Diagnostic criteria used for LVV	PET analysis	Threshold used for diagnosis of LVV at PET	Follow-up interval PET (months)	Diagnostic criteria	Authors	Year
GCA	P	35	N/A	GC	TAB, baseline, PET, clinical data, lab	QA: visual uptake intensity, TVS	Decrease in vessel uptake, TVS,	3 and 6	Clinical data, lab	Blockmans et al. [59]	2006
GCA	R	9	N/A	GC	Clinical data, lab	QA: visual SQA: vessel SUV_max_, vessel/liver SUV_max_ ratio	Decrease in: Vessel /liver SUV ratio cutoff 1	3	Clinical data, lab	Bertagna et al. [97]	2010
LVV	R	13	13	GC	Clinical data, lab	QA: visual uptake intensity, TVS SQA: vessel SUV_max_ CT: W, W/R	Decrease in TVS, W and W/R	NR	Clinical data, lab	Muto et al. [98]	2014
GCA and PMR	R	5	N/A	MTX	NR	QA: visual uptake intensity, vessel to liver uptake, TVS, TJS	Decrease in: TVS and TJS	Median 10.7	clinical data, lab	Camellino et al. [99]	2010
GCA, TA	R	10	N/A	CYC	NR	QA: visual vessel to liver uptake	Decrease in vessel uptake	3–4	Clinical data, BVAS, lab	Henes et al. [100]	2011
GCA, TA	R	5	N/A	GC	Clinical, lab, other imaging*	QA: vessel uptake intensity	Decrease in vessel uptake	Median 10	Clinical data, lab, other imaging	De Leeuw et al. [51]	2004
GCA and PMR	P	35	N/A		TAB, baseline, PET, clinical data, lab	QA: visual uptake intensity, TVS, TJS	Decrease in: vessel uptake, TVS and TJS	3 and 6	clinical data, lab	Blockmans et al. [60]	2007

N/A = not available

GC = glucocorticoids

CYC = cyclophosphamide

MTX = methotrexate

TVS = total vascular score

TJS = total joint score

BVAS = Birmingham vasculitis activity index

W = wall thickness

W/R = ratio of wall thickness to the radius

TAB = temporal artery biopsy

Asterisk (*) = CT angiography, magnetic resonance angiography (MRA), duplex ultrasound

A retrospective study by Bertagna et al. (Table 7) included a total of nine patients, with eight GCA patients having a normalized FDG-PET at follow-up after GC therapy, and one patient without any change in the FDG-PET [97]. Despite the small number of patients enrolled, the authors concluded that FDG-PET/CT might be a useful and accurate tool for evaluating disease progression.

In a study of five patients by Camellino et al. (Table 7), FDG-PET uptake decreased after the addition of methotrexate to the traditional GC treatment [99]. No studies have investigated whether GCA disease activity can be monitored by FDG-PET/CT in patients on GC-sparing drugs such as tumor necrotic factor α (TNF-α) blocking agents for TA or interleukin-6 receptor blockade (tocilizumab) for GCA

Interestingly, a recent abstract by Nielsen et al. [37] reported that the FDG-PET/CT score, based on the semiquantitative approach described by Meller et al. [55] (score < 3), remained positive for vasculitis after 3 days of GC treatment, but became negative after 10 days.

Recent studies have shown that at the temporal artery level, granulomatous infiltrates can persist even up to 1 year following the start of GC treatment [28, 101]. Macrophages and granulomatous inflammation have been reduced with GC treatment in experimental models [102], decreasing in a time-dependent manner from 78 to 100% at initial biopsy, to 50% at 9 months and 25% at 12 months in sequential temporal artery biopsies. Lymphocytes may persist longer [102], and have been reported to be present in GCA patients treated for up to 1 year [28].

This is in agreement with a study by Brack et al., in which macrophages persisted in the vessel walls of severe combined immunodeficiency disorder (SCID) mice engrafted with TAB after 1 week of GC treatment [103]. These findings are also in line with the fact that FDG-PET/CT(A) shows arterial wall uptake after 6 months in treated patients, although the uptake is no longer diagnostic for vasculitis.

Prieto-González et al. prospectively assessed GC-induced changes in CTA findings of LVV in patients with GCA [39]. Forty patients with biopsy-proven GCA evaluated by CTA at diagnosis were prospectively followed and scheduled for a new CTA after approximately 1 year of treatment. Vessel wall thickening, diameter, and contrast enhancement of the aorta and its tributaries were evaluated. Results were compared with those obtained at the time of diagnosis. CTA was repeated for 35 patients after a median follow-up of 13.5 months (IQ 25–75% 12.4–15.8). Arterial wall thickening was still present in 17 patients (68% of the patients who initially had LVV). The number of affected segments and the wall thickness at various aortic segments were significantly decreased, and no patients developed new lesions, new aortic dilation, or an increase in previous dilation. Contrast enhancement disappeared in 15 (94%) of 16 patients in whom this finding could be assessed. Signs of LVV improve with treatment. While contrast enhancement resolves in the majority of patients, vessel wall thickening persists in two-thirds. However, the number of affected aortic segments, as well as the aortic wall thickness, decreases significantly.

For PMR, there is one study with sequential PET/CT, by Blockmans et al. (Table 7), using the same methodology as that for GCA. The authors found that vascular FDG uptake was present in 11 patients, with slight or moderate uptake at diagnosis in nine of 35 patients, and that the uptake decreased after 3 and 6 months [60]. At baseline, FDG uptake in the shoulders was present in all but two patients, and uptake was still present, although to a lesser extent, after 3 and 6 months of GC therapy. The same holds true for the hips and the spinous processes. No difference was found in the predictive value of FDG uptake at baseline and after 3 months at the shoulders, hips, or spinous processes between patients who experienced a relapse and those who did not.

The optimal length of time before performing FDG-PET in PMR after treatment with GC is unclear. A recent study by Palard-Novello et al. evaluated the use of FDG-PET/CT(A) for the assessment of tocilizumab as first-line treatment in patients with PMR [104]. They found that FDG uptake decreased significantly but moderately after tocilizumab therapy in PMR patients, and may reflect disease activity.

**Table:** 

Consensus statement
FDG-PET/CT(A) may be of value for evaluating response to treatment by monitoring functional metabolic information and detecting structural vascular changes (evidence level III, grade C), but additional prospective FDG-PET/CT(A) studies are warranted.

## Consensus statements on open issues for future research agenda

### Clinical issues

- Further establish the role of FDG-PET/CT in patient management and evaluate its role in treatment monitoring. When to use FDG-PET/CT in the diagnosis, in the follow-up, and how often?
- Development of guidelines in LVV and PMR imaging with FDG-PET/CT(A) similar to those previously developed for FDG-PET/CT in oncology (EARL) criteria [26]. Randomized prospective studies are needed for more evidence.
- Including imaging biomarkers with the current diagnostic criteria to be considered for TA, GCA, and/or PMR.
- Finding a consensus in the clinical support for performing imaging as early as possible and before starting GC therapy if treatment delay can be justified due to non-critical symptoms.
- Further investigation of the GC effect on vascular FDG uptake.
- Theranostics (diagnostics for selected therapy) for LVV/PMR, which may open more paths to targeted therapy, resulting in personal/precision medicine. Radiolabeled tocilizumab or other monoclonal antibody PET tracers are potential candidates for this.
- Circumstances of when there may be myocardial involvement in patients with LVV should be further investigated (additional myocardial perfusion imaging, CT coronary calcium assessment, and CT angiography may be needed), including the risk of cardiovascular events due to drug therapy in LVV [105].
- Worldwide reimbursement for application of FDG-PET/CT(A) in LVV/PMR is needed.

### Methodological issues

- Standardization of visual scoring and (semi)quantification in FDG-PET in LVV and PMR is essential for interpretation, for optimal comparison among centers, especially in future multicenter trials.
- Decide how much thickening is mild, moderate, or severe (not established in literature). Based on our expertise, we think that ≥2 mm (up to 2.9) may be mild, ≥ 3 mm (up to 3.9) moderate, and ≥4 mm severe.
- Consensus needed on which quantification method to apply in LVV.
- An uptake interval of 60 min after FDG injection is recommended, but 90–120 min intervals can be evaluated for better image quality.
- Dual-time-point imaging may improve the target-to-background ratio, resulting in better image quality due to greater FDG blood pool clearance, particularly in patients with reduced kidney function. However, evidence-based data are lacking.
- New techniques for imaging and reconstruction of the skull that enable visualization of the superficial temporal artery, which will result in better comparison of local LVV with TAB.
- Value of combining FDG-PET with CTA as a standard procedure in LVV and PMR, single modalities or hybrid.
- Value of FDG-PET/MRI in monitoring LVV and PMR, i.e. reduction of radiation dose [106].
- Development of online training modalities for interpretation.

### Technical issues

- Optimization of the application of hybrid imaging in monitoring (residual) vascular wall disease in LVV.
- The use of vasculitis-specific tracers, directed against cells/proteins involved in and unique for the pathophysiology of LVV and PMR, should be investigated.
- New developments in camera systems, such as PET/MRI, enable us to combine metabolism or other molecular targets (PET) with vascular tissue layer characterization (MRI), including a reduction in radiation dose and improved cranial visualization. The value of these new multi-modality imaging systems may be of interest for LVV assessment and monitoring.
- Optimal use of (low-dose) CT to distinguish active atherosclerosis from active vasculitis by pattern recognition, visually as well as with the use of dedicated software methodologies (textural feature).

Several open issues are also in line with the recommendation for the use of imaging in large vessel vasculitis in clinical practice of the European League Against Rheumatism (EULAR) [107].

## Conclusion

The present procedural recommendation paper provides recommendations to assist imaging specialists and clinicians in requesting, performing, and interpreting the results of FDG-PET in patients with suspected LVV and PMR.

Based on the present clinical data, FDG-PET/CT(A) has an important role in the diagnosis of extracranial vascular involvement in patients with LVV/PMR, but additional randomized studies are needed to support this.

Improvements in FDG-PET/CT(A) procedures will help to optimize the diagnostic and monitoring value of this technique in LVV/PMR.

Visual qualitative methods are most commonly used, but semiquantitative methods such as the vascular/blood ratio and vascular/liver ratio using SUVs are increasingly being used.

The addition of CTA to FDG-PET provides high-resolution imaging of vascular morphology that can potentially improve diagnostic accuracy, but more importantly provides information on the presence of possible complications such as stenosis, organ ischemia, aneurysm formation, and dissection.

Further prospective studies involving large cohorts of GCA/PMR patients are needed to investigate and validate the role of semiquantitative methods for the assessment of LVV.

Several other open issues, as stated above, need to be studied for optimal performance of FDG-PET/CT(A) in the diagnosis, (treatment) monitoring, and future theranostics in LVV/PMR, further improving the levels of evidence and grades of recommendations.

## Electronic supplementary material

ESM 1 (DOCX 16 kb)
