# Supplementary material for: FDG-PET/CT(A) imaging in large vessel vasculitis and polymyalgia rheumatica: joint procedural recommendation of the EANM, SNMMI, and the PET Interest Group (PIG), and endorsed by the ASNC
Source: Eur J Nucl Med Mol Imaging. 2018 Apr 11;45(7):1250–69. doi: 10.1007/s00259-018-3973-8 (PMC5954002; doi:10.1007/s00259-018-3973-8)
Supplement: Supplementary file 1 — (DOCX 16 kb) [file 259_2018_3973_MOESM1_ESM.docx]

**Supplementary appendix**

**Supplement 1:** Levels of evidence and grades of recommendations [107].

| **Rating**  Level | **Description** |
| --- | --- |
| I | Evidence obtained from meta-analysis of multiple well-designed, controlled studies; randomized studies with low false-positive and low false-negative errors (high power) |
| II | Evidence obtained from at least one well-designed experimental study; randomized studies with high false-positive and/or false negative errors (low power) |
| III | Evidence obtained from well-designed quasi experimental studies (e.g. nonrandomized controlled single-group, pre-post, cohort, time, or matched case-control studies) |
| IV | Evidence from well-designed non experimental studies (e.g. comparative and correlational descriptive and case studies) |
| V | Evidence from case reports and clinical examples |

| **Grade** |  |
| --- | --- |
| A | Evidence of type I or consistent findings from multiple studies of types II, III or IV |
| B | Evidence of II, III or IV; findings are generally consistent |
| C | Evidence of II, III or IV; findings are inconsistent |
| D | Little or no systematic empiric evidence |

**Supplement 2:** Search strategy and selection criteria**.**

We describe the different methods used to perform FDG-PET/CT in LVV, including PMR and the role of FDG-PET/CT in the diagnosis of LVV/PMR, assessment of response to therapy, as well as introducing a proposal to standardize image interpretation criteria. Literature search has been performed through PubMed database (search date: from inception to 01.03.2017) using the following key words combination: ((PET) OR (positron emission tomography) OR (computed tomography) OR (imaging) OR (FDG) OR (fluorodeoxyglucose)) AND ((sensitivity) OR (specificity) OR (accuracy) OR (diagnosis) OR (response) OR (monitoring)) AND ((Takayasu) OR (giant cell) OR (polymyalgia) OR (vasculitis) OR (aortitis) OR (arteritis)). Only articles in English language were selected. Small case series were excluded.

To inform our Review, we asked expert in the field to identify articles using the authors’ own file system, on the topics addressed in this paper. The final reference list was generated on the basis of originality and relevance to the broad scope of this Review. This Review and the recommendations on the use of FDG-PET/CT(A) were developed by an interdisciplinary panel of experts on FDG-PET/CT in LVV/PMR. Expert consensus was used to propose recommendations in the absence of sufficiently robust data. Levels of evidence and grades of recommendations were attributed to the different indications according to published criteria (Supplement 1). The paper was drafted and circulated among all panel members followed by subsequent rounds of revisions until consensus was achieved.
